# The Relationship between Marital and Sexual Satisfaction among Married Women Employees at Golestan University of Medical Sciences, Iran

**Published:** 2014

**Authors:** Tayebe Ziaee, Yadollah Jannati, Elham Mobasheri, Taraneh Taghavi, Habib Abdollahi, Mahnaz Modanloo, Naser Behnampour

**Affiliations:** 1 Assistant Professor, Counseling and Reproductive Health Research Centre, Golestan University of Medical Sciences, Gorgan, Iran.; 2 Assistant Professor, Psychiatry and Behavioral Sciences Research Center, Addiction Institute AND Department of Psychiatric Nursing, School of Nursing and Midwifery, Mazandaran University of Medical Sciences, Sari, Iran.; 3 Associate Professor, Department of Gynecology, School of Medicine, Golestan University of Medical Sciences, Gorgan, Iran; 4. Assistant Professor, Department of Psychiatric Nursing, School of Nursing and Midwifery, Tehran University of Medical Sciences, Tehran, Iran.; 5 Department of Medical-Surgical Nursing, School of Nursing and Midwifery, Golestan University of Medical Sciences, Gorgan, Iran.; 6 Assistant Professor, Department of Community and Mental Health Nursing, School of Nursing and Midwifery, Golestan University of Medical Sciences, Gorgan, Iran.; 7 Department of Health, School of Health, Golestan University of Medical Sciences, Gorgan, Iran.

**Keywords:** Enrich Marital Satisfaction Questionnaire, Married Women, Marital Satisfaction, Sexual Satisfaction

## Abstract

**Objective:** There are various elements affecting the healthy family such as marital satisfaction. Various factors such as sexual satisfaction have an important impact on satisfaction of marital relationship. The present study aimed to determine the association of marital satisfaction with sexual satisfaction among sexually active employee women.

**Methods:** This analytical descriptive study was carried on 140 married women employed at educational and medical centers of Golestan University of Medical Sciences. Questionnaires for data collection included Enrich Marital Satisfaction Questionnaire and self-constructed questionnaire (demographic characteristic and sexual satisfaction). Data were analyzed using descriptive statistics, χ^2^ and Spearman statistical test. Statistical significant level was set as 0.05.

**Results: **The findings showed that in marital satisfaction scale, the majority of the participants (63.6%) were very satisfied and none of them were very unsatisfied. In sexual satisfaction scale, most of the participants (56.4%) expressed extremely satisfaction rate and only 0.7% were not satisfied with their sexual relationship. Marital satisfaction was significantly associated with sexual satisfaction (p ≤ 0.001). So with the increase of sexual satisfaction, there was an increase in marital satisfaction accordingly. The findings indicated that there was a significant association between sexual satisfaction and age (p = 0.086). Level of education was associated significantly with the marital satisfaction (p = 0.038). The effects of sexual satisfaction on marital satisfaction were moderated by number of children and the level of education.

**Conclusion:** The findings have implications for improving of couples' marital satisfaction by highlighting the need for awareness of sexual quality. According to the findings, it seems that development of educational programs and pre-marriage counseling is necessary. Continuous education would be helpful after marriage in addressing couples' unique transitional needs in marital life.

## Introduction

No society can claim to be healthy unless it has healthy families. Marriage and marital relationship is the start of forming a family (1, 2). Marriage has been documented in every known culture. More than 90% of the world's population will marry at least once (3). In literature marriage is described as a normative, personal life event in adulthood and involves the cohabitation of two people with different characteristics and needs (4). 

In general, people get married for specific purposes such as finding meaning in life and loving for a better quality of marital life. It is worth noting that continuation of marriage may depend on factors like marital relationship; because matrimony is more successful when spouses establish a sense of satisfaction with each other (5, 6).

Marital relationship is defined in terms of one or many of the following variables: marital satisfaction, dyadic adjustment, and communication or conflict resolution style. Marital satisfaction is simply the degree of contentment regarding certain aspects of marital relationship as well as the whole relationship. According to some previous researches, marital satisfaction refers to a subjective and global evaluation of the relationship (7, 8). It is a situation in which, couples have satisfaction and feel happy and living together (9).

The concept of marital satisfaction is a multifaceted and multidimensional concept including psychological, socioeconomic and spiritual components. Measures of marital satisfaction, in fact, vary from one researcher to another and their operational definition of marital satisfaction. In particular, the criteria for a satisfying marital relationship may be highly varied and may depend on a unique set of culturally enforced norms, obligations, and values (6, 10).

Indeed, marital satisfaction is influenced by many factors, for example, safe and pleasurable sexual relationship is mentioned to be one of the most important factors noticed in many researches (11). 

The research findings indicate that sexual affairs are at first rank in a married life and sexual satisfaction can bring about a favorable and convenient marriage (12). 

Sexuality is one of the most complex and important aspects of women's life. Sexual satisfaction has been defined as "an effective response arising from one’s subjective evaluation of the positive and negative dimensions associated with one’s sexual relationship" (13, 14). 

Although a few studies have been conducted on Iranian population to elucidate detailed information concerning marital dissatisfaction, sexual dissatisfaction underpins the most cause of marital dissatisfaction in Iran (70%) (11). 

The nature of satisfactory things may change due to passage of time and some demographic factors such as age at marriage, number of children, religious beliefs, and health status (12, 13). Other demographic characteristics such as the quality of marriage and sexual satisfaction are related to marital satisfaction, as well (13, 15). 

Despite the abundance of research in this area, rarely investigations have been conducted on Iranian couples. In general, research studies have focused on marital satisfaction and sexual satisfaction separately on specific groups (women with breast cancer, middle-aged couples and couples with infertility problems) (16-19). Most studies investigated whether demographic variables and intrapersonal and interpersonal factors (i.e. relationship quality, religious orientation, gender power, stress, quality of life, mental health problems, chronic disease, age difference of couples, child raising styles and children's educational achievement) were associated with marital or sexual satisfaction independently (4, 8, 11, 12, 18, 20-23). Previous study was reported the association between sexual and marital satisfaction but there is no evidence for moderating effects of these factors which have an impact on relationship between sexual satisfaction and marital satisfaction.

This research would be helpful to identify some factors affecting on marital satisfaction. Improving this awareness may persuade healthcare providers to develop context-based program in addressing marital satisfaction, thereby, higher marital satisfaction may lead to family stability.

The present study aimed to determine the association of marital satisfaction with sexual satisfaction (pre-, during and post-sexual intercourse) among sexually active employee woman.

## Materials and Methods

This analytical descriptive study was carried on married women employed at educational and medical centers of Golestan University of Medical Sciences in Gorgan (Panj-e-Azar, Taleghani, and Deziyani Hospital). 140 eligible women voluntary agreed to participate in the study, who were selected through purposive sampling, in regard to inclusive and exclusive criteria. 

These women had been married for at least one year and were living with their husbands, and were sexually active and were on their first marriage at the time of data collection. The participants, who were known as cases of psychiatric and physical disease and who were divorced or widow, were not eligible to participate in this study and were excluded. According to the finding of previous study, the correlation coefficient between sexual satisfaction and marital satisfaction was 0.46 (24). In this study, the sample size was estimated 152 (r_0 _= 0.2, α = 0.05, β = 0.05). 155 questionnaires were distributed among eligible participants, 12 women did not fulfill the questionnaire and three of uncompleted questionnaire were discarded. Although the response rate was not higher than in most studies on sexual satisfaction, we could not exclud the possibility that those who refused to fulfill the questionnaire had more or less sexual satisfaction than those who replied. 

Data collection was carried out with three questionnaires including demographic questionnaire, Enrich Marital Satisfaction Questionnaire and self-constructed Sexual Satisfaction Questionnaire. Prior to beginning the study, participants were informed about the general objective and the voluntary and confidential nature of participation. Because of the sensitivity of research topic, researchers in person distributed the questionnaire privately among the participants, who were interested in taking part in this research. After fulfilling the anonym questionnaires, they returned it through a postage-paid envelop. A cover letter explained the objective of the study and participants were requested not to write their names and addresses to insure the information confidentiality. 

A demographic questionnaire was used to gain pertinent information regarding participants' age, employment status, marital status, income, number of children, length of marriage, educational status and ethnicity, as well as their husbands' age, educational status and ethnic background. 

The rate of marital satisfaction was measured by Enrich Marital Satisfaction Questionnaire. It consists of 47 items, all items have to be answered on a 5-point Likert scale (1 = totally agree, 2 = agree, 3 = not agree not disagree, 4 = disagree, and 5 = totally disagree). Scores can range from 47 to 235, with higher scores indicating greater marital satisfaction. The Persian form of this instrument is valid among Iranian population. It was tasted and the Cronbach's alpha of the questionnaire was 0.95 (25). Marital satisfaction was considered into six following categories; very unsatisfied, unsatisfied, satisfied, relative satisfied, very satisfied and extremely satisfied. 

The Sexual Satisfaction Questionnaire is a 20-item self-construct questionnaire measuring the rate of sexual satisfaction before, while and after sexual intercourse. Women rate each question on a 4-point scale: most of the time, sometimes, seldom, or never; these are scored 1, 2, 3, and 4, respectively. Total scores can be obtained by summing relevant items. Scores can range from 20 to 80; higher scores reflect women who were more satisfied in their sexual activity. Sexual satisfaction was classified into four following categories; not satisfied, relative satisfied, very satisfied, and extremely satisfied. Reliability of self-construct Sexual Satisfaction Questionnaire was established using a pilot test by collecting data from 30 married female employees at a Golestan University of Medical Sciences which not included in the sample. According to the nature of data which was ordinal, to assess reliability of questionnaire, Cronbach's alpha was used (α = 0.759). 

Then the collected data were analyzed by using SPSS for Windows 0.16 (SPSS Inc., Chicago, IL, USA) and using descriptive statistics, chi-square test and Spearman statistical test. Significant level was set as 0.05. The liner regression was used to explore a model for predicting the marital satisfaction as the dependent variable.

## Results

This finding showed that the mean age of participants was 34.89 (±6.99) (rang of 28 years) and the average age of their husbands was 38.12 (±7.42) (varying from 24 to 54 years). Most of the participants (79.24%) had bachelor degrees or higher. Length of marriage was 10 (±6.75) (ranging from 1 to 25 years). Among the participants, 44.2% had two or more children and 28.8% did not have any children. Most of the participants' ethnicity was Fars (97%); which was as the same as their husbands' (98.6%), as a result we did not consider it in relation to marital and sexual satisfaction.

In marital satisfaction scales, the majority of the participants (63.6%) had a high satisfaction and none of them (n = 0) were extremely unsatisfied. 

In sexual satisfaction scale, 56.4% of the participants expressed an extremely satisfied rate and only 0.7% were not satisfied with their sexual relationship. The result from correlation between marital and sexual satisfaction indicates that marital satisfaction was significantly associated with sexual satisfaction (χ^2^ = 46.37, p ≤ 0.001). So with the increase of marital satisfaction, sexual satisfaction also increased. 

From 75% of those who reported "relative satisfied" in their marital relationship or had a "very or extremely satisfaction", only 25% had lower satisfaction in their sexual relationship (in 35-49 rate) and 88.7% reported "extremely satisfied" in their sexual relationship. Although 8.1% of participants who were "very unsatisfied" in their marital relation, none of them reported "extremely satisfied" in their sexual relation. Descriptive statistics and correlation matrix for sexual and marital satisfaction are shown in table 1.

The mean score for sexual satisfaction was 64.57 (±9.74). The analysis of variance (ANOVA) test comparing the mean age of participants in different levels of sexual satisfaction indicated that lower mean age led to increase in sexual satisfaction and this relationship was almost significant (p = 0.086). 

The findings indicated that there was not any statistical significant association between sexual satisfaction and length of marriage, number of children, level of education, and age difference of couples. Although, the higher level of education, the lower number of children, shorter duration of marriage and lower age difference of couples increase the sexual satisfaction, the association was not statistically significant.

The mean score for marital satisfaction was 167.64 (±31.53). The findings showed that marital satisfaction significantly associated with the level of education (p = 0.038), the higher the level of education, the higher the level of marital satisfaction. Although, age, length of marriage, number of children, and age difference of couples were not significant for predicting marital satisfaction, on the whole, the maritally more satisfied participants were those who reported as having a lower age, length of marriage, number of children, and age difference of couples. The result of regression analysis indicated that the magnitude of marital satisfaction can be predicted from the sexual satisfaction level based on following model:

The linear regression model of correlation of marital and sexual satisfaction

Marital satisfaction = 48.595 + 1.846 × sexual satisfaction (p = 0.001)

Although the increase in the number of children can reduce the rate of both sexual and marital satisfaction, it can increase the association between sexual and marital satisfaction. The level of education can increase this relationship accordingly as well. Other observed demographic variables did not have impact on association between sexual and marital satisfaction (Table 2).

**Table 1:** Correlation of marital satisfaction and sexual satisfaction based on their categories

**Marital satisfaction** **Sexual satisfaction**	**Very unsatisfied** **(≥ 122)**	**Unsatisfied** **(123-147)**	**Relative satisfied** **(148-202)**	**Very/extremely satisfied** **(203-228)**			**Total**
**Not/relative satisfied (35-49)**	6 (50%)	03 (25%)	3 (25%)	0 (0%)		12 (100%)	
**Very satisfied (50-64)**	4 (9.8%)	10 (24.4%)	26 (63.4%)	1 (2.4%)		41 (100%)	
**Extremely satisfied (65-80)**	0 (0%)	08 (11.3%)	49 (69%)	14 (19.7%)		71 (100%)	
**Total**	10 (8.1%)	21 (16.9%)	78 (62.9%)	15 (12.1%)	124 (100%)		

**Table 2:** The effect of age and level of education on correlation of marital and sexual satisfaction

		**N (%)**	**Sexual satisfaction (Mean)**	**Marital satisfaction (Mean)**	**Coefficient**	**P-value**
**Number of children**	0	029 (21.80)	67.27	178.52	0.390	0.0660
	1	043 (32.33)	64.25	165.19	0.514	0.0010
	2 ≥	062 (45.86)	64.22	166.22	0.605	0.0001
**Educational status**	Diploma or less	029 (20.86)	62.53	164.12	0.534	0.0060
	Bachelors or higher	110 (79.24)	65.13	168.53	0.547	0.0001

## Discussion

This research revealed that more than half of the participants (56.4%) were extremely satisfied with their sexual relationship; only 0.7% was not satisfied. Auslander found that 15% of adolescents and young adults reported feeling of satisfaction in their sexual relationships (26). Ji et al. found that 98.8% of married couples (98.7% of the married women) were satisfied, fairly satisfied or very satisfied with their sexual relationships (13). But Lau found that 38.3% of women in China were satisfied with their sexual life in the last 12 months (21). Comparison of the finding shows that adolescents and younger adults had lower rate of satisfaction in their sexual activities, regardless of marital status. According to Rezaipour et al. there was a statistical significant association between reach orgasm and sexual satisfaction and 80.8% of participants had orgasm and men reported a higher proportion of satisfaction than women (27). Although the mean score sexual satisfaction in our study was lower than this study, comparison between the rate of satisfaction in men and women may identify the cause of difference.

In marital satisfaction scores, the mean score for marital satisfaction was 167.64 (±31.53). The majority of the participants (63.6%) had a very satisfaction and none of them (n = 0) were very unsatisfied. According to finding in Iran, the mean score for marital satisfaction was 156.10 (±19.77) (27), and there was a statistical significant difference between marital satisfaction of employed women and housewives [175.17 (± 24.47) vs. 162.97 (±26.81), respectively; p < 0.01], other researchers found that only 14% of participants were very satisfied and 69% were relatively satisfied in their marital relation (2). Despite the fact that the overall satisfaction was lower than our finding, finding of another study discriminated that the mean score of participants who were employed, was higher than housewives.

The results revealed that marital satisfaction was significantly related to sexual satisfaction, this finding was also supported by some other researchers, as they notified that some aspect of sexual satisfaction have considerable impact on marital satisfaction (9, 11, 27-31).

Based on the findings, a significant reverse association was observed between age and sexual satisfaction, finding of some studies support this finding that the lower the age of participants, the more the sexual satisfaction (11, 27, 32).

The results of this study showed that there was no statistical significant association between sexual satisfaction and some related factors such as age, age difference of couples, length of marriage, and number of children. 

Increase in the level of education was associated with increase in the rate of sexual satisfaction. Conversely, decrease in age, age difference of couples, length of marriage, and number of children were associated with increase in the rate of sexual satisfaction. 

The results of other studies indicated that sexual satisfaction was associated with some factors such as the length of marriage, age difference of couples (11, 27), number of children and level of education (13, 27). Ji et al. found a positive correlation between education and sexual satisfaction. Ji and Norling argue that education can affect the economic stability. When couples are educated they have a greater chance of achieving economic stability, and thus, higher marital and sexual satisfaction than couples with less formal education (13). 

The results of this study indicated that marital satisfaction was associated with some related factors such as age, age difference of couples, length of marriage, and number of children. Although the lower of age, length of marriage, number of children, age difference of couples and the higher marital satisfaction, the difference was not statistically significant. Only level of education was significantly related to marital satisfaction. Pervious study had also confirmed this finding; however, their results were limited by male participants (28). However, there is some discrepancy between this research and other studies (1).

A recent study by Rahmani et al. showed the significant association between age difference of couples and marital satisfaction in women (11). The finding of another researcher confirmed this result and this finding demonstrated that the rate of marital satisfaction in age groups who had less than three years age difference, was higher than others (1).

The findings of this study indicated that marital satisfaction was not significantly associated with number of children. However, many studies have rejected this finding of the present study (1, 9, 25, 33). 

This study found that the less the length of marriage, the more marital satisfaction exists among the spouses. Although the association was not statistically significant, many studies have confirmed the finding of this study (1, 9, 28, 34, 35). The finding of this research contradicts those of Jalili's study; he believes that husband's cooperation in house work and his sympathy may increase due to passage of time after marriage. In addition, they gradually learn love making and the changes can lead to increase marital satisfaction (33).

The finding implied that lower mean age led to increase in marital satisfaction, although this relationship was not significant. However, there are some congruencies between this and other studies (1, 9, 32). Tahmasebi et al. found that marital satisfaction had a positive correlation with level of education and age difference of couples among housewives (2). 

Carlson believes that growing older leads to a change in the person's expectations and attitude. Hence, age can have a great influence on marital satisfaction, while Nasehi found that there was no statsitically significant correlation between age and marital satisfaction in women (1). 

In conclusion, findings of this study indicated that prediction of marital satisfaction by some demographic variables is possible. It is important to consider several limitations and future directions of the present research when interpreting these findings. The differences between our results and other research findings may be due to the lack of variety in sampling. Our study analyzed only women who were employees of healthcare system. Since the knowledge of staff who working in health system is more than other people in society/population, these results may not be readily generalized to other samples of women who may differ in age, occupation, health literacy, and socio-economic status. In addition, because most of the participants were highly educated, the results of this study are not representative of all women. 

Further researches must be done with national random samples and both partners simultaneously to elucidate the impact of more socio-cultural factors on sexual and marital satisfaction. According to finding, increase in sexual satisfaction has a positive effect on marital satisfaction, so it seems giving information about improving sexual activity skills and marital relationship in pre-marriage consultation is very important for the quality and stability of family basis. In conclusion, the health planners and the officials in charge pay more attention to women's sexual problems and sexual counseling clinics must be established in healthcare centers. In addition to our recommendation, further research involving a more diverse sample (e.g. gender, employment status and field of work) should be conducted to determine whether these findings are generalizable across the society.

***Limitations and Future Research***

The results of this study should be viewed in consideration of several limitations. First, this is a correlational study, and the authors cannot infer cause and effect. Second, self-report questionnaires were used in this study that may contain errors. Finally, the participants in this study were married employed women, which limit the generalizability of the findings to other groups of populations. 

## Footenote

**Declaration of interest:** None.
